# Clinical outcome of primary giant cell tumor of bone after curettage with or without perioperative denosumab in Japan: from a questionnaire for JCOG 1610 study

**DOI:** 10.1186/s12957-018-1459-6

**Published:** 2018-08-08

**Authors:** Hiroshi Urakawa, Tsukasa Yonemoto, Seiichi Matsumoto, Tatsuya Takagi, Kunihiro Asanuma, Munenori Watanuki, Akira Takemoto, Norifumi Naka, Yoshihiro Matsumoto, Akira Kawai, Toshiyuki Kunisada, Tadahiko Kubo, Makoto Emori, Hiroaki Hiraga, Hiroshi Hatano, Satoshi Tsukushi, Yoshihiro Nishida, Toshihiro Akisue, Takeshi Morii, Mitsuru Takahashi, Akihito Nagano, Hideki Yoshikawa, Kenji Sato, Masanori Kawano, Koji Hiraoka, Kazuhiro Tanaka, Yukihide Iwamoto, Toshifumi Ozaki

**Affiliations:** 1 Department of Orthopaedic Surgery, Nagoya University, 65 Tsurumai, Showa-ku, Nagoya, Aichi 466-8550 Japan; 2 Division of Orthopaedic Surgery, Chiba Cancer Center, Chiba, Japan; 3 Department of Orthopaedic Surgery, Cancer Institute Hospital, Japanese Foundation for Cancer Research, Tokyo, Japan; 4 Department of Orthopaedic Surgery, Juntendo University, Tokyo, Japan; 5 Department of Orthopaedic Surgery, Graduate School of Medicine, Mie University, Tsu, Japan; 6 Department of Orthopaedic Surgery, Tohoku University Hospital, Sendai, Japan; 7 Department of Orthopaedic Surgery, School of Medicine, Yokohama City University, Yokohama, Japan; 8 Musculoskeletal Oncology Service, Osaka International Cancer Institute, Osaka, Japan; 9 Department of Orthopaedic Surgery, Kyushu University, Fukuoka, Japan; 10 Department of Orthopaedic Surgery, National Cancer Center, Tokyo, Japan; 11 Department of Medical Materials for Musculoskeletal Reconstruction, Graduate School of Medicine, Dentistry, and Pharmaceutical Sciences, Okayama University, Okayama, Japan; 12 Department of Orthopaedic Surgery, Hiroshima University, Hiroshima, Japan; 13 Department of Orthopaedic Surgery, Sapporo Medical University, Sapporo, Japan; 14 Department of Orthopaedic Surgery, Hokkaido Cancer Center, Sapporo, Japan; 15 Department of Orthopaedic Surgery, Niigata Cancer Center Hospital, Niigata, Japan; 16 Department of Orthopaedic Surgery, Aichi Cancer Center, Nagoya, Japan; 17 Department of Orthopaedic Surgery, Kobe University, Kobe, Japan; 18 Department of Orthopaedic Surgery, Kyorin University, Mitaka, Japan; 19 Department of Orthopaedic Surgery, Shizuoka Cancer Center, Shizuoka, Japan; 20 Department of Orthopaedic Surgery, Gifu University, Gifu, Japan; 21 Department of Orthopaedic Surgery, Osaka University, Osaka, Japan; 22 Department of Orthopaedic Surgery, Teikyo University, Tokyo, Japan; 23 Department of Orthopaedic Surgery, Oita University, Oita, Japan; 24 Department of Orthopaedic Surgery, Kurume University, Kurume, Japan; 25 Department of Endoprosthetic Surgery, Oita University, Oita, Japan; 26 Kyushu Rosai Hospital, Kitakyushu, Japan; 27 Department of Orthopaedic Surgery, Graduate School of Medicine, Dentistry, and Pharmaceutical Sciences, Okayama University, Okayama, Japan

**Keywords:** Giant cell tumor of bone, Outcome, Denosumab, Japan

## Abstract

**Background:**

Giant cell tumor of bone (GCTB) is an intermediate tumor known to be locally aggressive, but rarely metastasizing. To plan a prospective study of GCTB, we performed a questionnaire survey for institutions participating in the Bone and Soft Tissue Tumor Study Group (BSTTSG) in the Japan Clinical Oncology Group (JCOG) in 2015.

**Methods:**

We reviewed 158 consecutive patients with primary GCTB treated with curettage without perioperative denosumab from 2008 to 2010 in Japan. We investigated local and distant recurrence rates after definitive curettage. We also investigated the recurrence rate after treatment with preoperative and/or postoperative denosumab with curettage in recent years. There were 40 patients treated with perioperative denosumab, and the factors affecting recurrence in them were investigated.

**Results:**

Answers were available from 24 of 30 institutions (80.0%) participating in JCOG BSTTSG. Thirty (19.0%) and 4 (2.5%) of 158 patients developed local and distant recurrence after curettage without perioperative denosumab from 2008 to 2010, respectively. Campanacci grade and embolization before surgery were significantly associated with increasing incidence of local recurrence after curettage (*p* = 0.034 and *p* = 0.022, respectively). In patients treated with perioperative desnosumab, 120 mg denosumab was administered subcutaneously for a median 6 (2–41) and 6 (1–14) times in preoperative and postoperative settings, respectively. The recurrence rates were 6 of 21 (28.6%), 2 of 9 (22.2%), and 0 of 10 (0.0%) in the preoperative, postoperative, and both pre- and postoperative denosumab treatment groups, respectively. With all of the preoperative treatments, administration exceeding five times was significantly associated with a decreased incidence of local recurrence after curettage (*p* < 0.001).

**Conclusion:**

The recurrence rate of GCTB was still high after curettage, especially in Campanacci grade III, and improvements in the therapeutic strategy are needed in this cohort. There is a possibility that a sufficient dose of preoperative denosumab can reduce recurrence after curettage. Recently, we have started a clinical trial, JCOG1610, to investigate the efficacy of preoperative denosumab in patients who can be treated with curettage in GCTB.

## Background

Giant cell tumor of bone (GCTB) is an intermediate tumor known to be locally aggressive, but rarely metastasizing in the WHO classification [1]. GCTB possibly originates from the metaphyseal region [2], and accounts for 4–5% of all skeletal neoplasms in Japan. Local and distant recurrence rates were reported in 24.8–30.8% [3–6] and 2% [7, 8] of the patients after curettage, respectively. To reduce local recurrence and preserve the adjacent joint, adjuvant treatments such as high-speed burr [3], phenol [5, 9], ethanol, liquid nitrogen [5], and polymethyl methacrylate (PMMA) [3, 5, 9] have been reported. Even though there were some reports of GCTB in Japan [7, 10, 11], the recent clinical results of GCTB after curettage in multiple institutions in Japan have not been well documented.

Denosumab is a fully human monoclonal antibody that inhibits the receptor activator of NF-κB (RANK) ligand (RANKL) and then interrupts RANK-RANKL interactions. In GCTB, the stromal cells and osteoclast-like giant cells express RANKL and RANK, respectively, and the RANK-RANKL interaction is considered to be necessary for the differentiation and activation of osteoclasts [12]. Therefore, the RANK-RANKL interaction has a critical role for bone destruction in GCTB, and dramatic change was observed after treatment of denosumab in GCTB. Multinucleated osteoclast-like giant cells and stromal cells were decreased after denosumab treatment for GCTB [13]. A recent phase 2 study demonstrated the effects of denosumab for patients with unresectable GCTB and salvageable GCTB whose surgery was associated with severe morbidity [14]. Denosumab was accepted for health insurance coverage in Japan in 2014. However, the role of denosumab in patients with GCTB who can be treated by curettage has not been well defined.

We performed a questionnaire survey for institutions participating in the Bone and Soft Tissue Tumor Study Group (BSTTSG) in the Japan Clinical Oncology Group (JCOG) in 2015 for planning a clinical trial of JCOG1610, a randomized phase III study of preoperative denosumab with curettage for GCTB. The first aim of the present study was to identify the historical outcome after curettage for GCTB without perioperative desnosumab in Japan. The second purpose was to identify the clinical use of perioperative denosumab and the factors influencing local recurrence after perioperative denosumab with curettage.

## Methods

### Patients

We reviewed 158 patients with GCTB treated by curettage without perioperative desnosumab from 2008 to 2010 in institutions participating in the JCOG BSTTSG. We also reviewed 40 patients with GCTB treated with curettage and perioperative denosumab.

### Methods

We performed a questionnaire survey for institutions participating in the BSTTSG in JCOG in April and June 2015. This questionnaire survey was performed for planning a clinical trial of JCOG 1610 (UMIN000029451), a randomized phase III study of preoperative denosumab with curettage for GCTB. We retrospectively reviewed clinical records and filled out the questionnaire. The questionnaire included standard treatments (e.g., local adjuvant, reconstruction) of curettage for GCTB in each institution, details (e.g., number of extremities, Campanacci grade, pathological fracture at presentation, and embolization before surgery) of GCTB treated with curettage from 2008 to 2010, and clinical results (e.g., number of local recurrences, distant recurrences, and death) after the curettage, and details (e.g., sites, Campanacci grade, pathological fracture at presentation, time to the recurrence, final joint preservation, and embolization before surgery) of the patients with local recurrence. We also asked about perioperative use of denosumab for GCTB. The questionnaire included the indications of denosumab for GCTB in each institution, the number of patients treated with preoperative, postoperative, and both pre- and postoperative denosumab, respectively, number of times of perioperative denosumab administration, and clinical results of local recurrence after the perioperative denosumab with curettage. This study was approved by the ethics committee of Nagoya University Graduate School and School of Medicine (Nagoya, Japan) and a waiver of informed consent was provided.

### Statistics

A chi-square test was used to analyze the correlation of various clinical factors with recurrence. Clinical factors such as sites (extremity, trunk), Campanacci grade (I, II, III), pathological fracture at presentation (yes, no), timing of denosumab (preoperative only vs postoperative only, both preoperative and postoperative vs preoperative or postoperative, preoperative only vs both preoperative and postoperative, postoperative only vs both preoperative and postoperative), and number of times of denosumab administration (5>, 5≦) were analyzed as related to the frequency of recurrence. *p* values of < 0.05 were considered significant. Statistical analysis was done using IBM SPSS Statistics 24.0 software (IBM, Armonk, NY, USA).

## Results

Responses to the questionnaire were available from 24 of 30 institutions (80.0%) participating to JCOG BSTTSG. Standard treatments of curettage for GCTB in the 24 institutions are summarized in Table 1. As a local adjuvant therapy, high-speed burr was used after curettage in 22 of 24 (92%) institutions followed by ethanol (8 of 24 institutions, 33%), liquid nitrogen (6 of 24 institutions, 25%), and phenol (3 of 24 institutions, 13%). Autologous bone graft and polymethyl methacrylate (PMMA) were used for reconstruction after curettage in 18 (75%) and 17 (71%) of 24 institutions, respectively. Characteristics of the patients with GCTB were summarized in Table 2. Primary tumor sites were in an extremity in 151 of 158 (96%) patients and trunk in 7 of 158 (4%). Sixteen of 158 (10%) evaluated as Campanacci grade I, 97 of 158 (61%) as Campanacci grade II, and 45 of 158 (29%) as Campanacci grade III.

**Table 1:** Standard treatments with curettage for GCTB^a^ in 24 institutions

Treatments	No. of institutions (%)
Local adjuvants	
High-speed burr	22 (92%)
Ethanol	8 (33%)
Liquid nitrogen	6 (25%)
Phenol	3 (13%)
Reconstruction after curettage	
Autologous bone graft	18 (75%)
PMMA^b^	17 (71%)
β-TCP^c^	11 (46%)
Hydroxyapatite	10 (42%)
Allogeneic bone graft	2 (8%)

^a^*GCTB* giant cell tumor of bone

^b^*PMMA*, polymethyl methacrylate

^c^*β-TCP*, β tricalcium phosphate; some of the replies by responded institutions are overlapped

**Table 2:** Characteristics of patients of GCTB^a^ treated with curettage from 2008 to 2010

Characteristics	No. of patients (%)
Primary tumor site	
Extremity	151 (96%)
Trunk	7 (4%)
Campanacci grade	
Grade I	16 (10%)
Grade II	97 (61%)
Grade III	45 (29%)
Pathological fracture at presentation	
Yes	26 (16%)
No	132 (84%)
Do you use denosumab for this case now?	
Yes	83 (52%)
No	75 (48%)
Embolization before surgery	
Yes	8 (5%)
No	150 (95%)

^a^*GCTB*, giant cell tumor of bone

Thirty of 158 (19.0%) developed local recurrence, and 4 of 158 (2.5%) developed distant recurrence after curettage without perioperative denosumab. In extremities, 29 of 151 (19.2%) developed local recurrence, and 4 of 151 (2.6%) developed distant recurrence after curettage. There were no deaths after curettage. Campanacci grade and embolization before surgery were significantly associated with an increased incidence of local recurrence after curettage (*p* = 0.034 and *p* = 0.022, respectively) (Table 3). Demographics of local recurrent patients after curettage for GCTB were summarized in Table 4. Local recurrence occurred in 1 of 16 (6.2%) in Campanacci grade I, 15 of 97 (15.5%) in Campanacci grade II, and 14 of 45 (31.1%) in Campanacci grade III. Median time to local recurrence was 15.5 months (5–69 months) after curettage, and joint preservation was achieved in 26 of 30 patients (86.7%).

**Table 3:** Univariate analysis of local recurrence in patients with GCTB^a^ treated with curettage from 2008 to 2010. (*n* = 158)

Clinical factors	No. of local recurrence (%)	Univariate analysis
		*p* value^b^
Site		
Extremity	29/151 (19.2%)	*p* = 0.746
Trunk	1/7 (14.3%)	
Campanacci grade		
Grade I	1/16 (6.3%)	*p* = 0.034
Grade II	15/97 (15.5%)	
Grade III	14/45 (31.1%)	
Pathological fracture at presentation		
Yes	5/26 (16.7%)	*p =* 0.972
No	25/132 (18.9%)	
Do you use denosumab for this case now?		
Yes	15/83 (18.1%)	*p =* 0.758
No	15/75 (20.0%)	
Embolization before surgery		
Yes	4/8 (50%)	*p =* 0.022
No	26/150 (17.3%)	

^a^*GCTB*, giant cell tumor of bone

^b^Chi-square test

**Table 4:** Characteristics of local recurrent patients after curettage for GCTB^a^ from 2008 to 2010

Sites	Campanacci grade	Pathological fracture at presentation	Time to recurrence (months)	Final joint preservation	Embolization before curettage
Tibia	II	No	25	Possible	No
Femur	II	No	60	Possible	Yes
Metatarsal bone	II	No	60	Impossible	Yes
Femur	III	No	6	Impossible	No
Tibia	III	No	36	Possible	Yes
Tibia	II	No	27	Possible	No
Fibula	II	No	6	Possible	No
Femur	III	No	11	Possible	No
Femur	II	No	28	Possible	No
Ulna	III	No	6	Possible	No
Femur	II	No	9	Possible	No
Tibia	II	No	21	Possible	No
Femur	II	No	24	Impossible	No
Femur	III	No	6	Possible	No
Radius	II	No	6	Impossible	No
Tibia	III	Yes	24	Possible	Yes
Tibia	III	Yes	14	Possible	No
Fibula	II	No	35	Possible	No
Femur	III	Yes	21	Possible	No
Lumbar spine	III	No	60	Not available	No
Femur	II	No	12	Possible	No
Femur	III	Yes	5	Possible	No
Femur	II	No	6	Possible	No
Talus	II	No	12	Possible	No
Femur	III	Yes	30	Possible	No
Femur	III	No	6	Possible	No
Femur	III	No	22	Possible	No
Femur	III	No	7	Possible	No
Femur	II	No	17	Possible	No
Femur	I	No	7	Possible	No

^a^*GCTB*, giant cell tumor of bone

The indications of denosumab for GCTB at the 24 institutions are summarized in Table 5. As a general policy, denosumab was used perioperatively at 6 of 24 (25%) institutions. Actually, 40 patients were treated with perioperative denosumab in 16 institutions. The number of GCTB patients treated with perioperative denosumab and that of the institutions where they were treated were listed in Table 6. Denosumab was administered subcutaneously at 120 mg, but the dosing interval was not included in the questionnaire. Median number of times of denosumab administration were 6 (2–41) and 6 (1–14) in the preoperative and postoperative settings, respectively. The local recurrences were observed in 6 of 21 (28.6%), 2 of 9 (22.2%), and 0 of 10 (0.0%) patients treated with the preoperative, postoperative, and both preoperative and postoperative denosumab, respectively. In 31 patients treated with any preoperative denosumab, administration exceeding 5 times was significantly associated with a decreased incidence of local recurrence after curettage (*p* < 0.001) (Table 7). Question for toxicity or side effects during perioperative denosumab were not included in the questionnaire survey.

**Table 5:** Indication of denosumab for GCTB^a^ in 24 institutions

Indications of denosumab	No. of institutions (%)
Unresectable	21 (88%)
Difficult to joint preservation	17 (71%)
Expanding to soft tissue	11 (46%)
Adjusting the time of operation	7 (29%)
Perioperative use with curettage	6 (25%)
No experience of denosumab use	3 (13%)

^a^*GCTB*, giant cell tumor of bone; some of the replies by responded institutions are overlapped

**Table 6:** No. of patients and institutions treated with perioperative denosumab for GCTB^a^

Timing of perioperative denosumab	No. of patients (%)	No. of institutions (%)
Preoperative only	21 (53%)	10 (63%)
Postoperative only	9 (23%)	5 (31%)
Both pre- and postoperative	10 (25%)	5 (31%)
Total	40	16^*^

^a^*GCTB*, giant cell tumor of bone

*Some institutions performed denosumab at different timing

**Table 7:** Univariate analysis of local recurrence in patients treated with perioperative denosumab and curettage for GCTB^a^ (*n* = 40)

Comparison of factors	No. of local recurrence (%)	Univariate analysis
		*p* value^b^
All patients (*n* = 40)		
Timing of denosumab		
Preoperative only	6/21 (28.6%)	*p* = 0.719
Postoperative only	2/9 (22.2%)	
Timing of denosumab		
Both pre- and postoperative	0/10 (0.0%)	*p =* 0.068
Pre- or postoperative only	8/30 (26.7%)	
Preoperative (*n* = 31)		
Times of denosumab administration		
5>	5/7 (71.4%)	*p <* 0.001
5≦	1/24 (4.2%)	
Timing of denosumab		
Preoperative only	6/21 (28.6%)	*p =* 0.060
Both pre- and postoperative	0/10 (18.9%)	
Postoperative (*n* = 19)		
Times of denosumab administration		
5>	0/7 (0.0%)	*p =* 0.253
5≦	2/12 (16.7%)	
Embolization before surgery		
Postoperative only	2/9 (22.2%)	*p =* 0.115
Both pre- and postoperative	0/10 (0.0%)	

^a^*GCTB*, giant cell tumor of bone

^b^Chi-square test

## Discussion

To plan a clinical trial JCOG 1610, a randomized phase III study of preoperative denosumab with curettage for GCTB, we conducted a questionnaire survey to comprehend the historical clinical results after curettage of GCTB without perioperative denosumab. Although the clinical outcomes after curettage of GCTB have been reported sporadically in Japan [7, 10, 11], the more recent clinical results of GCTB after curettage in multiple institutions in Japan are not as clear. To determine the recent perioperative use in Japan, we also reviewed patients with GCTB treated with curettage and perioperative denosumab. Even though denosumab was accepted for health insurance coverage in Japan in 2014, the risk/benefit ratio of denosumab when used for patients with GCTB who are treatable by curettage is not well defined.

There are some limitations in our study. First, because of its questionnaire format, we did not have data regarding the follow-up period after curettage. We investigated GCTB patients treated from 2008 to 2010 and performed this questionnaire survey in 2015, meaning that the follow-up period can be considered adequate given that most recurrences in GCTB occur within 5 years [3–6] and recurrent GCTB is usually treated at the same institution where the first surgery was performed. Second, there was a lack of important data such as size of tumor, detailed sites, and Campanacci grade of GCTB treated with perioperative denosumab, which could act as confounding factors. Because our study is a questionnaire survey, we could not conduct an additional survey due to unlinkable anonymizing of our data. Third, we could not perform multivariate analysis because of the small number of recurrences and lack of information regarding other important clinical factors. Finally, we could not determine whether the patients treated with preoperative denosumab were all suitable for curettage from the time of their initial consultation.

As a local adjuvant therapy, a high-speed burr was used after curettage in 92% of institutions followed by ethanol (33%), liquid nitrogen (25%), and phenol (13%) in our study. Clinical results of these local adjuvant therapies have been reported [3, 5, 9], but it is difficult to determine the advantage of each treatment. Generally, the high-speed burr is easier to use than drug therapies such as ethanol, liquid nitrogen, and phenol, accounting for its extensive use in Japan.

In our study, autologous bone graft and PMMA were used for reconstruction after curettage in 75% and 71% of institutions, respectively. Some reports demonstrated the clinical benefit of PMMA for decreasing local recurrence after curettage of GCTB [3, 4, 6, 9]. Autologous bone graft is widely used in Japan because the lack of a bone bank precludes routine use of allogenic bone.

In our study, the local recurrence rate after curettage was 19.0%. Previous reports showed local recurrence of GCTB in 24.8–30.8% after curettage [3–6], and so our local recurrence rate is slightly better than that documented in these previous reports. Past reports have shown some clinical factors affecting local recurrence such as tumor extension, surgical margin, local adjuvant therapy, Campanacci grade, use of PMMA, and soft tissue progression on multivariate analyses [4, 5, 9]. In our study, Campanacci grade was well balanced similar to previous studies [9, 15], and we ascribe the better local recurrence rate achieved in our study to the wide use of local adjuvant treatments as well as PMMA in many institutions.

In our study, Campanacci grade was significantly associated with increasing incidence of local recurrence after curettage, and this result was similar to that noted in previous studies [4]. Embolization before surgery was also significantly associated with the increasing incidence of local recurrence after curettage, but it was difficult to interpret. Since the embolization is usually used for patients with GCTB which is large and expected bleeding, there was a possibility that these factors had affected the result. However, in the present study, one of the recurrent cases after embolization had GCTB in metatarsal bone (Table 4). Our study included only a small number of cases of GCTB in the trunk and a previous report showed a high local recurrence rate of 43.3% in axial cases after curettage [4]. However, there was no difference in the local recurrence rate between location in the trunk (1 of 7 patients, 14.3%) and extremity (29 of 151 patients, 19.2%) in our study. There was no significant relation between pathological fracture at first visit and local recurrence in our study. A past report also could not demonstrate an effect of pathological fracture on local recurrence in a meta-analysis [16]. Local recurrent GCTB is known to be highly re-recurrent after curettage with rates of re-recurrence of 32 to 34% [17, 18]. Our study included no patients after recurrence, and our analysis was limited to primary tumors. Distant recurrence rate was 2.6% after curettage in our study, and this result was similar to that noted in previous studies [7, 8].

In our study, 16 of 24 (67%) institutions actually performed perioperative use of denosumab with curettage, and the recurrence rates were 6 of 21 (28.6%), 2 of 9 (22.2%), and 0 of 10 (0.0%) with preoperative, postoperative, and both pre- and postoperative treatments with curettage, respectively. One study on the perioperative use of denosumab for GCTB with curettage demonstrated local recurrence in 17 of 116 patients (15%) with a median follow-up period of 13.0 months [19]. However, the study included patients with unresectable GCTB and salvageable GCTB whose surgery was associated with severe morbidity, and the effect of denosumab for patients with GCTB who can be treated by curettage at the first visit was not clear. Our study may have also included some patients who could not be treated by curettage at first, but we could not identify those patients due to the questionnaire format used.

Median numbers of administration times of denosumab were 6 (2–41) and 6 (1–14) in the preoperative and postoperative settings, respectively in our study. Some reports demonstrated histopathological changes after 6 months treatment with denosumab [20, 21], but 6 months are thought to be too long to use it as a post- and/or preoperative treatment in patients with GCTB which can be treated by curettage. In our study, more than five administration times was significantly associated with a decreased incidence of local recurrence after curettage in 31 patients treated with preoperative or both pre- and postoperative denosumab, meaning that a sufficiently high dose of preoperative denosumab can suppress local recurrence after curettage. When used as a running dose, five times administration of denosumab takes 3 months. This relatively short administration period is associated with major benefits, both economic and social, for patients, and this dose is specified in JCOG1610.

The clinical use of perioperative denosumab is complicated by various issues such as economic problem, side effects, and pregnancy. The cost of one-shot denosumab (120 mg) for GCTB is 46,685 yen (approximately 420 dollar) in Japan as of October 2017. In the previous phase 2 trial of GCTB, denosumab caused diverse side effects such as arthralgia (20%), headache (18%), nausea (17%), fatigue (16%), back pain (15%), extremity pain (15%), hypocalcemia (5%), and osteonecrosis of jaw (1%) of any grade [14]. The use of denosumab for pregnant women should be avoided because it was reported to increase postnatal mortality, decreased body weight gain, and decreased growth/development in a study of infants exposed in utero in cynomolgus monkeys [22]. This may affect the clinical use of denosumab for premenopausal women. In addition, there is a report that denosumab treatment in postmenopausal women with osteoporosis did not interfere with fracture healing [23], but the effects of denosumab on pathological fracture healing and final joint preservation have not been well understood in GCTB patients. Malignant transformation occurs in less than 1% of GCTB [1], and recently, malignant transformation was reported after treatment with denosumab [24] and requires particular caution. In addition, recent study showed a higher rate of recurrence in the GCTB treated with denosumab and curettage compared to historical control without denosumab in retrospective study [25]. For these reasons, perioperative treatment of denosumab should not be done unless an advantage is considered or proved in GCTB which can be treated by curettage.

At present, we have started a clinical trial, JCOG1610 (UMIN000029451), to investigate the efficacy of preoperative denosumab in patients with GCTB which can be treated with curettage. The primary aim of JCOG1610 is to confirm the effects of preoperative denosumab on recurrence after curettage. A previous report demonstrated that proliferation of stromal cells cultured from clinical specimens following denosumab treatment was approximately 50% slower than that of specimens from untreated patients [20]. Even though denosumab did not completely prevent proliferation of stromal cells which have been considered as genuine tumor cells [20], there is a possibility that preoperative denosumab may decrease local and distant recurrences after the curettage of the tumor stromal cells biologically suppressed by denosumab. Secondary endpoints of JCOG1610 include overall survival, joint-preserved survival, local relapse-free survival, metastasis-free survival, adverse events, serious adverse events, surgical and postoperative complications, and discontinuance of denosumab. Systemic denosumab treatment can affect joint-preserved survival, and both local and distant recurrence.

## Conclusions

In conclusion, the recurrence rate of GCTB after curettage was 19.0% in Japan and especially high in Campanacci grade III; therefore, improvements in the therapeutic strategy are needed in this cohort. There is a possibility that a sufficient dose of preoperative denosumab can reduce recurrence after curettage. Recently, we have started JCOG1610 to investigate the efficacy of preoperative denosumab in patients with GCTB which can be treated with curettage.

## Abbreviations

BSTTSG: Bone and Soft Tissue Tumor Study Group
GCTB: Giant cell tumor of bone
JCOG: Japan clinical oncology group
PMMA: Polymethyl methacrylate
RANK: Receptor activator of NF-κB
RANKL: Receptor activator of NF-κB ligand
